# Artificial intelligence-based modeling and validation for prediction of drug delivery capacity and cytotoxicity in design of porous materials

**DOI:** 10.3389/fchem.2026.1824825

**Published:** 2026-05-14

**Authors:** Sameer Alshehri, Mahboubeh Pishnamazi

**Affiliations:** 1 Department of Pharmaceutics and Industrial Pharmacy, College of Pharmacy, Taif University, Taif, Saudi Arabia; 2 Institute of Research and Development, Duy Tan University, Da Nang, Vietnam; 3 School of Engineering and Technology, Duy Tan University, Da Nang, Vietnam

**Keywords:** extreme gradient boosting, histogram-based gradient boosting, metal organic framework, modeling, principal component analysis

## Abstract

Metal organic frameworks (MOFs) have attracted attention for application of drug delivery because of their ordered porous properties. Optimizing both drug loading capacity and biocompatibility remains a complex challenge for MOFs because these performance indicators depend on nonlinear interactions among structural, compositional, and physicochemical features. In this study, an explainable ensemble learning framework was developed to predict Drug Loading Capacity (g/g) and Cell Viability (%) of drug-loaded MOFs using curated structural descriptors and experimentally derived data. A structured preprocessing pipeline—including robust scaling, multicollinearity control, target-guided encoding, and exploratory dimensionality analysis—was implemented prior to model training. Three gradient boosting algorithms, Gradient Boosting Trees (GBT), Extreme Gradient Boosting (XGBoost), and Histogram-Based Gradient Boosting (HGB), were systematically evaluated. Among them, HGB demonstrated superior predictive performance, achieving test R^2^ values of 0.9924 for drug loading capacity and 0.9987 for cell viability, with minimal generalization gap between training and testing datasets. An ablation study confirmed that model performance arises from the synergistic contribution of preprocessing strategies and boosting architecture. Furthermore, SHAP and LIME analyses provided both global and local interpretability, revealing chemically meaningful feature contributions and enhancing model transparency. The results demonstrate that explainable gradient boosting models can reliably capture complex structure–property relationships in MOF systems, offering a robust and interpretable computational tool to accelerate data-driven optimization of drug delivery platforms while reducing reliance on extensive experimental screening.

## 1 Introduction

Metal–organic frameworks (MOFs) are crystalline hybrid compounds built from metal centers bonded to organic linkers, creating porous three-dimensional structure with adjustable physicochemical characteristics to make them suitable for various applications. Their structural modularity enables precise control over pore size, surface functionality, topology, and chemical environment, making them particularly suitable for drug delivery applications where the release of drug can be controlled by interactions with the chemical structure of MOF (Filiz et al., 2025; Hamadi et al., 2025). However, optimizing MOF-based drug carriers requires simultaneous consideration of multiple interdependent variables, including metal identity, ligand composition, surface charge, particle size, and drug–framework interactions (Jin et al., 2026; Menon and Fairen-Jimenez, 2025; Shi et al., 2026). These variables collectively influence both drug loading capacity and cytotoxic response, generating highly nonlinear structure–property relationships that are difficult to capture using conventional linear modeling approaches (Qin et al., 2026).

Mechanistic modeling and first-principles models can be employed for evaluation of interactions between MOF structure and the drug molecules (Valenzuela, 2025). Considering a wide range of drugs and their assessment for delivery and controlled release by MOFs, implementing computational models based on molecular modeling is challenging and needs huge resources and time to find the solution. As such, models based on data-driven are preferred for this application, as these models can be run for a large number of entities with great accuracy (Huwaimel and Alqarni, 2025). Some information data from the chemistry of MOFs can be collected along with drugs data to build such models and generalize them.

From a data-driven perspective, predicting MOF performance involves structured datasets characterized by heterogeneous features, moderate dimensionality, potential multicollinearity, and mixed numerical–categorical attributes. Traditional regression techniques often struggle under such conditions due to linearity assumptions and sensitivity to correlated predictors (Barman and Sarkar, 2025a). Ensemble learning methods, particularly gradient boosting algorithms, provide a flexible alternative by constructing additive models that iteratively minimize prediction error through nonlinear tree-based learners. These models inherently capture higher-order feature interactions without explicit feature engineering and incorporate regularization mechanisms to improve generalization performance (Artrith et al., 2021; Barman and Sarkar, 2025b; Tao et al., 2025). The main properties of MOFs which should be predicted via data-driven models are loading capacity of MOFs as well as their cytotoxicity for use in drug delivery. Qin et al. developed data-driven models for evaluation of MOFs properties in drug delivery and it was revealed that ML (Machine Learning) models are superior in analysis of MOFs (Qin et al., 2026).

Despite their predictive strength, boosting algorithms are frequently criticized for limited interpretability. In materials informatics and pharmaceutical design, transparency is essential to ensure that predictions remain chemically and biologically plausible. Accordingly, explainable artificial intelligence (XAI) techniques have emerged as complementary tools for quantifying prediction behavior. This study integrates robust preprocessing strategies, ensemble gradient boosting models, and *post hoc* interpretability analysis to develop a reliable predictive framework for simultaneous estimation of drug loading capacity and cell viability in drug-loaded MOFs. By coupling statistical rigor with algorithmic efficiency and interpretability, the proposed methodology aims to support data-driven optimization of MOF-based drug delivery systems.

## 2 Data set used for model development

The dataset analyzed here was obtained from the source (Wang et al., 2024) which reports an extensive collection of drug-loaded MOFs considering wide range of MOFs and drugs molecules. The data comprises complete records with no missing value; therefore, no imputation or data reconstruction procedures were necessary (Qin et al., 2026). The final curated dataset provided in (Wang et al., 2024) was used directly for model development and evaluation. The same data has been used and analyzed in previous research with focus on machine learning, and their methods are applied in this study to build new models of machine learning (Huwaimel and Alqarni, 2025; Qin et al., 2026).

Two response variables were modeled in this study which are related to MOFs and drugs, i.e., Cell Viability (%) (containing 444 samples) and Drug Loading Capacity (g/g) (containing 161 samples). These outputs capture, respectively, the biological compatibility of the drug-loaded framework and its capacity to encapsulate therapeutic agents—two performance criteria that often exist in delicate balance.

Beyond the metal nodes, the composition and functional groups of organic ligands play a crucial role in modulating drug–carrier interactions. Specific molecular substructures affect adsorption behavior, intermolecular interactions, and cytotoxic responses (Qin et al., 2026).

**FIGURE 1:**
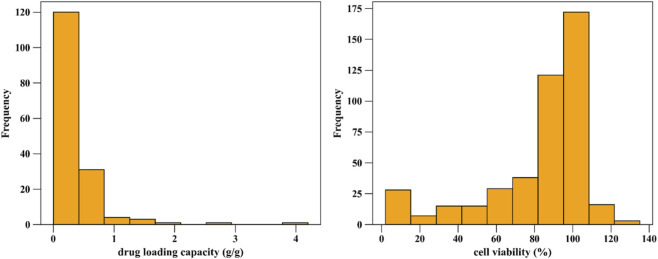
Histograms of drug loading capacity (g/g) and cell viability (%) for the studied drug-loaded MOFs dataset.

Collectively, the dataset integrates compositional, structural, and physicochemical descriptors that are mechanistically relevant to both drug loading and biocompatibility, providing a robust foundation for predictive modeling. The distribution patterns of the two response variables are illustrated in Figure 1, showing a positively skewed distribution for drug loading capacity and a wider variability in cell viability values. Qin et al. (2026) reported the same analysis of this data.

### 2.1 Pre-processing

Prior to model development, a structured pre-processing pipeline was implemented to enhance statistical stability and predictive reliability. As such, the dataset of MOF was first partitioned into a training set and an independent test set to build ML models and tune them. All preprocessing operations, including robust scaling, multicollinearity control, target-guided encoding, and dimensionality analysis, were fit exclusively on the training portion of dataset. The learned scaling parameters and encoding statistics were subsequently applied to the test set. During cross-validation, this procedure was repeated within each training fold, such that no information from the validation or test targets contributed to the preprocessing or model fitting steps, thereby preventing data leakage.

Continuous variables were standardized using Robust Scaling, in which features are centered by the median and scaled according to the interquartile range (IQR). This approach reduces sensitivity to extreme observations while preserving distributional structure, making it suitable for physicochemical descriptors that may exhibit skewness or non-Gaussian behavior (Panjei et al., 2022).

To address potential redundancy among correlated physicochemical attributes, multicollinearity analysis was conducted using correlation screening and Variance Inflation Factor (VIF) evaluation. Highly collinear predictors were removed or consolidated to prevent instability in model estimation and inflated variance, thereby improving interpretability and generalization performance (Dormann et al., 2013; Senter, 2008).

The distribution of the response variables—Cell Viability (%) and Drug Loading Capacity (g/g)—was examined for skewness. Where necessary, variance-stabilizing transformations (e.g., logarithmic or Box–Cox transformation) were considered to improve normality and modeling efficiency (Box and Cox, 1964).

Finally, the categorical MOF identity variable was encoded using target-guided encoding, which replaces categories with statistically derived representations based on their relationship with the response variable. This method preserves predictive signal while avoiding the dimensional expansion associated with one-hot encoding, particularly when multiple framework types are present (Micci-Barreca, 2001). Together, these steps ensure a balanced treatment of structural, compositional, and physicochemical descriptors while promoting robust and interpretable predictive modeling.

## 3 Methodology

The overall methodological framework adopted in this study follows a structured and sequential workflow designed to ensure robust predictive modeling and interpretability. Beginning with curated MOF structural and physicochemical descriptors, the dataset undergoes systematic preprocessing, including scaling, multicollinearity assessment, categorical encoding, and exploratory dimensionality analysis. The processed features are then utilized to train three ensemble learning algorithms—GBT, XGBoost, and HGB.

**FIGURE 2:**
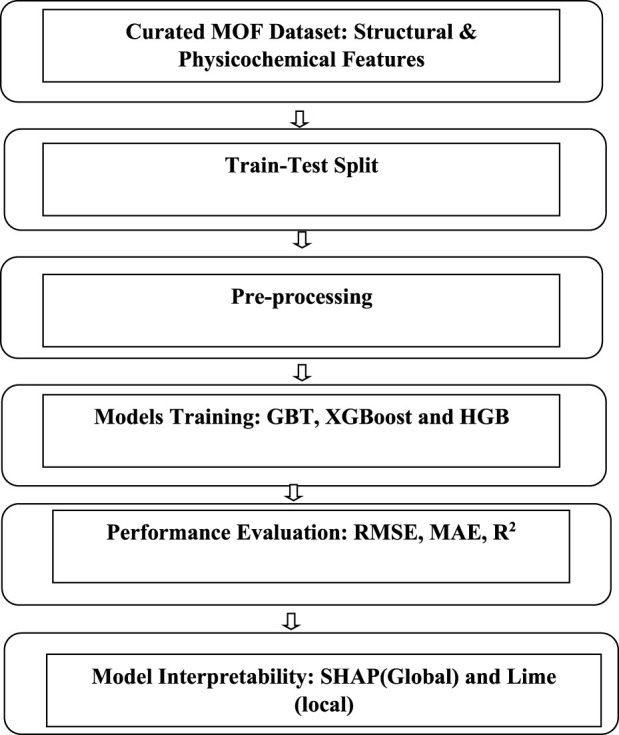
Overall proposed method for MOF data analysis.

Finally, model interpretability is investigated through global and local explanation techniques using SHAP and LIME to elucidate feature contributions and ensure transparency in prediction behavior. The complete methodological pipeline is illustrated in Figure 2.

### 3.1 PCA for dimensionality reduction

To further investigate the intrinsic structure of the feature space and assess potential redundancy among descriptors, PCA was employed as an exploratory dimensionality reduction technique. In this work, PCA was used only for exploratory analysis and sensitivity assessment, and that PCA-based models are not the models ultimately adopted for the results and comparisons discussed elsewhere in the manuscript. PCA converts the original correlated variables into a reduced set of orthogonal components, called principal components, that capture the greatest variance within the dataset (Abdi and Williams, 2010; Kurita, 2021). Thus, PCA was utilized as a complementary analytical tool for exploratory analysis rather than as a mandatory preprocessing step for predictive modeling.

### 3.2 Gradient boosting trees model

GBT is an ensemble technique that creates a robust predictive model by progressively merging several weak learners, usually in the form of decision trees. Unlike standalone trees, which may suffer from high variance or limited generalization, gradient boosting constructs trees in a stage-wise manner, where each new tree is trained to correct the residual errors of the previous ensemble (Natekin and Knoll, 2013). The approach is rooted in functional gradient descent, where the model minimizes a specified loss function by iteratively moving in the direction of the negative gradient.

Let $y_{i}$ denote the true response and $F_{m}\left(x_{i}\right)$ the prediction at iteration *m*. The model is updated as (Xi et al., 2018):

$$F_{m}\left(x\right)=F_{m-1}\left(x\right)+\eta h_{m}\left(x\right)$$

where $h_{m}\left(x\right)$ represents the newly fitted decision tree.

### 3.3 XGBoost model

XGBoost is an advanced implementation of gradient boosting that enhances predictive performance through regularization, parallel computation, and optimized tree construction. Like conventional GBT, XGBoost builds an additive ensemble of decision trees in a stage-wise manner (Ali et al., 2023). However, it incorporates a regularized objective function and second-order gradient information, leading to improved accuracy and computational efficiency (Chen et al., 2015).

At iteration *t*, the prediction is updated as:

$$\hat{y_{i}^{\left(t\right)}}=\hat{y_{i}^{\left(t-1\right)}}+f_{t}\left(x_{i}\right)$$

where $f_{t}$ represents the newly added regression tree. The objective function minimized at each step combines a differentiable loss function *L* and a regularization term Ω :

$$L^{\left(t\right)}=\sum_{i=1}^{n}L\left(y_{i},\hat{y}i^{\left(t\right)}\right)+\sum k=1^{t}\Omega \left(f_{k}\right)$$

The regularization component penalizes model complexity and is defined as:

$$\Omega \left(f\right)=\gamma T+\frac{1}{2}\lambda \sum_{j=1}^{T}w_{j}^{2}$$

where *T* is the number of leaf nodes, $w_{j}$ denotes the weight of leaf *j*, and γ and λ are regularization parameters controlling tree complexity and weight shrinkage, respectively.

### 3.4 HGB model

HGB is a more efficient version of gradient boosting that improves computational performance by converting continuous features into a set number of bins. Rather than considering every potential split value, the method organizes feature values into histograms and identifies the best splits within these discrete intervals (Guryanov, 2019). As in standard gradient boosting, the model is built sequentially. At iteration *m*, predictions are updated as:

$$F_{m}\left(x\right)=F_{m-1}\left(x\right)+\eta h_{m}\left(x\right)$$

The pseudo-residuals are computed as (Friedman, 2002):

$$r_{im}=-{\left[\frac{\partial L\left(y_{i},F\left(x_{i}\right)\right)}{\partial F\left(x_{i}\right)}\right]}_{F=F_{m-1}}$$

For regression tasks such as predicting drug loading capacity and cell viability, the squared error loss is commonly adopted (Guryanov, 2019; Tamim Kashifi and Ahmad, 2022).

### 3.5 Model training and hyperparameter configuration

To ensure optimal predictive performance, the hyperparameters of the ensemble learning models were tuned using cross-validation on the training dataset. The selected configurations were determined based on their ability to minimize prediction error while maintaining model generalization. The final hyperparameter settings used for training the Gradient Boosting Trees, Extreme Gradient Boosting, and Histogram-Based Gradient Boosting models are summarized in Table 1. These parameters were kept fixed during the final model training and evaluation stages.

**TABLE 1:** Hyperparameter configuration of the ensemble learning models used in this study.

Model	Hyperparameter	Value
GBT	Number of estimators	300
	Learning rate	0.05
	Maximum depth	4
	Minimum samples split	2
	Minimum samples leaf	1
	Subsample	0.9
XGBoost	Number of estimators	400
	Learning rate	0.03
	Maximum depth	5
	Subsample	0.8
	Colsample_bytree	0.8
	Gamma	0
	Regularization (lambda)	1
Histogram-based gradient boosting HGB	Learning rate	0.05
	Maximum iterations	350
	Maximum depth	6
	Minimum samples leaf	20
	L2 regularization	0.0
	Early stopping	Enabled

## 4 Results and discussion

The fitting accuracy of the developed ensemble models was assessed for both Drug Loading Capacity (g/g) as well as Cell Viability (%) (Huwaimel and Alqarni, 2025; Qin et al., 2026). In fact, two main variables were considered to assess the use of MOFs in drug delivery. MOFs with the highest loading capacity and least toxicity are preferred for drug delivery. Model accuracy was assessed using cross-validated R^2^ scores, independent test-set R^2^, RMSE, and MAE. Comparative results for all three algorithms—GBT, XGBoost, and HGB—are summarized in Tables 2–5. Overall, the models demonstrate strong predictive capability for both response variables, with HGB exhibiting the highest generalization performance across training and test datasets. Furthermore, to quantify uncertainty in model performance due to the relatively small test set (∼20% of the dataset), 95% confidence intervals for test R^2^ were estimated using bootstrap resampling (10,000 iterations) of the test predictions. The percentile method was used to obtain the confidence bounds.

**TABLE 2:** Coefficient of determination values of final models for drug loading capacity as output (80% of 161 samples were used for training and rest for testing).

Models	Mean CV R^2^ score	Train R^2^ score	Test R^2^ score	Test R^2^ (95% bootstrap CI)
GBT	0.9839 ± 0.0009	0.9787	0.9750	0.9638–0.9946
XGboost	0.9678 ± 0.0001	0.9798	0.9683	0.9488–0.9851
HGB	0.9990 ± 0.0003	0.9994	0.9924	0.9887–0.9996

**TABLE 3:** Error of final models for drug loading capacity (80% of 161 samples were used for training and rest for testing).

Models	RMSE		MAE	
	Train	Test	Train	Test
GBT	0.211	0.129	0.92	0.93
XGboost	0.171	0.090	0.88	0.89
HGB	0.146	0.076	0.68	0.59

**TABLE 4:** Coefficient of determination values of final models for cell viability (80% of 444 samples were used for training and rest for testing).

Models	Mean CV R^2^ score	Train R^2^ score	Test R^2^ score	Test R^2^ (95% bootstrap CI)
GBT	0.9490 ± 0.0041	0.9564	0.9409	0.9376–0.9722
XGboost	0.9516 ± 0.0002	0.9580	0.9486	0.9354–0.9743
HGB	0.9993 ± 0.0001	0.9995	0.9987	0.9802–0.9998

**TABLE 5:** Error of final models for cell viability (80% of 444 samples were used for training and rest for testing).

Models	RMSE		MAE	
	Train	Test	Train	Test
GBT	0.4669	0.6222	4.1420	5.7829
XGboost	0.4672	0.5136	3.863	3.980
HGB	0.4326	0.4652	3.402	3.521

Tables 2–5 collectively demonstrate the comparative predictive performance of the three ensemble learning algorithms for both Drug Loading Capacity and Cell Viability. For drug loading prediction, all models achieved high accuracy, with cross-validated and test R^2^ values exceeding 0.96, indicating strong generalization. To confirm generalization reliability, data splits were independently randomized, yielding consistent performance patterns. All preprocessing and encoding steps were restricted to the training data within cross-validation, eliminating risk of data leakage. Although external MOF datasets are currently unavailable for full validation, future study will incorporate independent data sources to further strengthen model assessment.

Among them, the HGB model delivered the best overall performance, achieving a test R^2^ of 0.9924 alongside the lowest RMSE and MAE values, suggesting minimal deviation between predicted and experimental values. GBT and XGBoost also performed competitively, though with slightly higher prediction errors. A similar trend is observed for cell viability prediction, where HGB again demonstrated superior performance with near-perfect agreement between training and test results (R^2^ = 0.9987) on the test set. While GBT and XGBoost achieved strong predictive capability (test R^2^ values above 0.94), their error metrics were comparatively larger. The consistently small gap between training and test metrics for HGB indicates stable learning behavior with limited overfitting. Overall, these results confirm that ensemble-based gradient boosting approaches are highly effective for modeling the nonlinear relationships between MOF structural descriptors and their physicochemical and biological responses, with the histogram-based variant offering the most robust generalization across both target variables.

**FIGURE 3:**
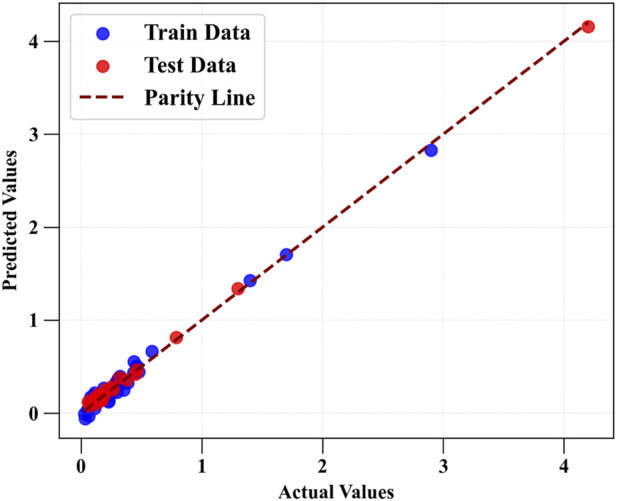
Comparison of estimated and source values for drug loading capacity using HGB.

**FIGURE 4:**
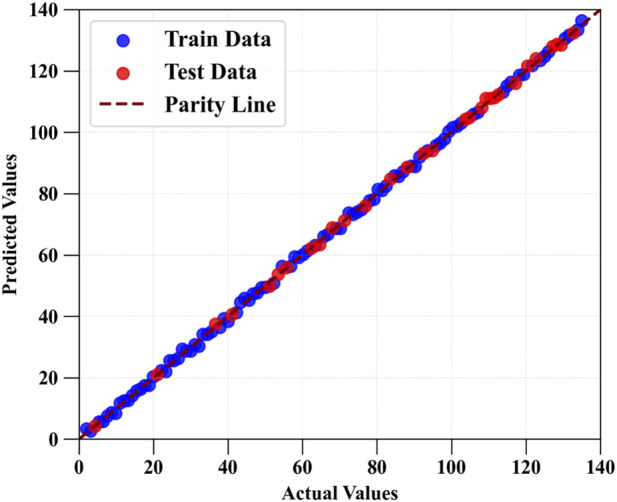
Comparison of estimated and source values for cell viability using HGB.

Figures 3, 4 present the parity plots comparing predicted and experimental values for Drug Loading Capacity and Cell Viability using the HGB model, identified as the best-performing algorithm (Qin et al., 2026). The tight clustering of both training and test samples around the reference line reflects high predictive accuracy and minimal systematic bias. Moreover, the absence of pronounced dispersion or structured deviation suggests that the model effectively captures the nonlinear relationships governing MOF structural features and their corresponding physicochemical and biological responses. Other works reported similar accuracy and trend for parity plots (Huwaimel and Alqarni, 2025; Qin et al., 2026).

**FIGURE 5:**
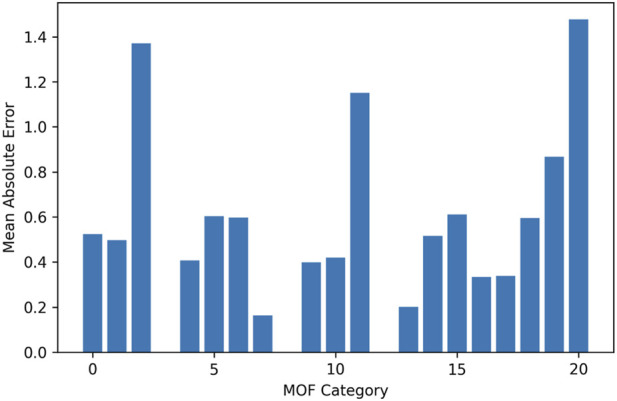
Drug loading: prediction error per MOF category.

**FIGURE 6:**
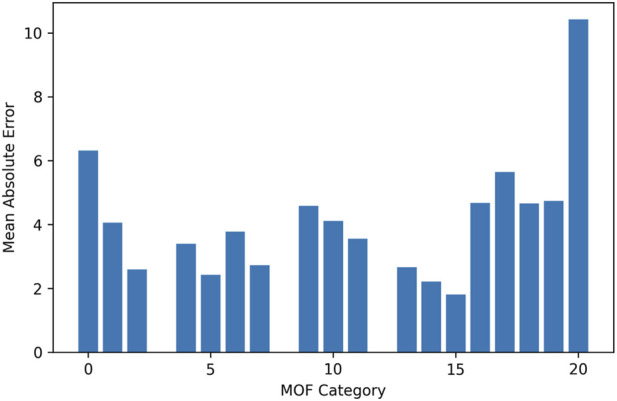
Cell viability: prediction error per MOF category.

Figures 5, 6 illustrate the distribution of prediction errors across different MOF categories for Drug Loading Capacity and Cell Viability, respectively. The plots reveal that the HGB model maintains consistently low absolute errors across most framework types, indicating stable performance irrespective of MOF identity. No single category exhibits disproportionately large deviations, suggesting that the model does not favor specific structural classes and generalizes well across chemically diverse frameworks. Minor variations in error magnitude among certain MOFs may reflect intrinsic structural complexity or limited representation in the dataset rather than systematic modeling bias. Overall, these results confirm the robustness and category-level reliability of the proposed predictive framework.

**FIGURE 7:**
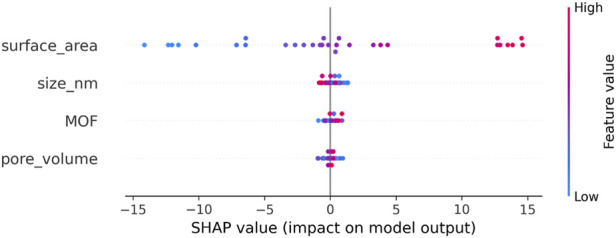
SHAP summary plot for drug loading capacity predictions.

**FIGURE 8:**
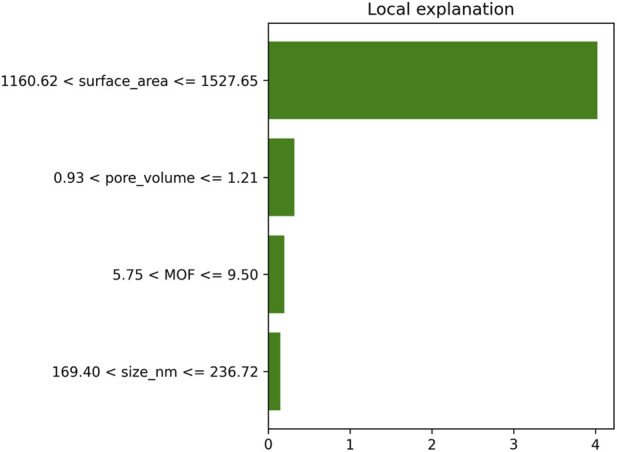
Lime local explanation drug loading capacity predictions.

Figures 7, 8 provide global and local interpretability analyses of the best-performing HGB model using SHAP and LIME, respectively. The SHAP summary plot (Figure 7) highlights the relative importance of structural and physicochemical descriptors in driving model predictions, revealing how variations in key features influence Drug Loading Capacity and Cell Viability across the entire dataset. The distribution of SHAP values indicates both the magnitude and direction of feature effects, offering a comprehensive understanding of dominant predictors. Complementarily, the LIME explanation in Figure 8 presents a localized interpretation for an individual prediction, illustrating how specific feature values contribute positively or negatively to the predicted outcome (Mohale and Obagbuwa, 2025). Together, these explainability tools enhance model transparency by linking predictive performance to chemically meaningful descriptors, thereby supporting the reliability and interpretability of the proposed framework.

Table 6 presents the ablation analysis conducted on the HGB model to evaluate the contribution of each preprocessing component to overall predictive performance. The full pipeline consistently achieved the highest accuracy for both Drug Loading Capacity and Cell Viability, confirming the effectiveness of the integrated methodological framework. Progressive removal of individual preprocessing steps resulted in measurable performance degradation, with the most pronounced decline observed when target-guided encoding was excluded. This highlights the critical role of chemically informed categorical representation in capturing MOF-specific effects. The omission of multicollinearity control and robust scaling also reduced predictive stability, though to a lesser extent, indicating their supportive yet meaningful contributions. In contrast, removing PCA-based exploratory screening produced only minor reductions in performance, consistent with its role as a structural analysis tool rather than a primary modeling requirement. Overall, the ablation results show that the reported predictive accuracy arises from the synergistic combination of preprocessing strategies and model architecture rather than from the boosting algorithm alone.

**TABLE 6:** Ablation study of the HGB model for both target variables.

Configuration	Drug loading test R^2^	Drug loading RMSE	Drug loading MAE	Cell viability test R^2^	Cell viability RMSE	Cell viability MAE
Full model (all steps)	0.9924	0.076	0.59	0.9987	0.4652	3.521
– Without robust scaling	0.9856	0.112	0.87	0.9918	0.8124	6.017
– Without multicollinearity removal	0.9813	0.135	1.04	0.9886	1.1243	8.445
– Without target encoding	0.9738	0.189	1.41	0.9762	2.4635	18.042
– Without PCA screening	0.9892	0.094	0.71	0.9971	0.5981	4.389

It is indicated that ensemble-based gradient boosting models are effective in capturing the complex, nonlinear relationships governing drug loading efficiency and cytotoxic response in MOF systems. Among the evaluated algorithms, the superior performance of the Histogram-Based Gradient Boosting model suggests that discretized split optimization combined with regularization provides enhanced generalization for structured physicochemical datasets. The minimal discrepancy between training and test metrics indicates stable learning behavior and limited overfitting, which is further supported by the ablation study confirming that predictive strength arises from the combined contribution of preprocessing strategies and model architecture. The interpretability analyses reinforce the chemical plausibility of the predictions, revealing that key structural descriptors and physicochemical attributes meaningfully influence both loading capacity and cellular viability. Importantly, the category-level error assessment demonstrates consistent performance across diverse MOF types, highlighting the robustness of the framework in handling structural heterogeneity. Collectively, these findings suggest that explainable ensemble learning approaches can serve as reliable predictive tools for accelerating MOF design and screening, reducing reliance on exhaustive experimental trials while maintaining interpretability grounded in chemically relevant features.

## 5 Conclusion

In this study, an explainable ensemble learning framework was developed to predict two critical performance indicators of drug-loaded metal–organic frameworks (MOFs): Drug Loading Capacity (g/g) and Cell Viability (%). By integrating structurally and chemically meaningful descriptors with a carefully designed preprocessing pipeline, three gradient boosting algorithms—GBT, XGBoost, and Histogram-Based Gradient Boosting (HGB)—were systematically evaluated. Among them, HGB demonstrated superior predictive performance, achieving near-perfect generalization with minimal overfitting across both target variables.

The ablation analysis confirmed that the strong predictive accuracy did not arise solely from the boosting architecture, but rather from the synergistic contribution of robust scaling, multicollinearity control, and chemically informed categorical encoding. Furthermore, global and local interpretability analyses using SHAP and LIME provided insight into the influence of structural and physicochemical features on model predictions, reinforcing the chemical plausibility and transparency of the proposed approach.

The consistent performance observed across different MOF categories highlights the robustness of the developed framework in handling structural diversity and nonlinear interactions. These findings demonstrate that explainable machine learning models can serve as reliable decision-support tools for accelerating MOF design and optimization. By reducing dependence on exhaustive experimental screening while maintaining interpretability, the proposed methodology contributes to the advancement of data-driven materials discovery in pharmaceutical and biomedical applications.

Future work may extend this framework to larger and more diverse datasets, incorporate external validation, and explore multi-objective optimization strategies for simultaneous enhancement of loading efficiency and biocompatibility.

## Data Availability

The original contributions presented in the study are included in the article/supplementary material, further inquiries can be directed to the corresponding author.
